# Towards an integrative approach to understanding collective behaviour in caterpillars

**DOI:** 10.1098/rstb.2022.0072

**Published:** 2023-04-10

**Authors:** Callum F. McLellan, Stephen H. Montgomery

**Affiliations:** School of Biological Sciences, University of Bristol, Bristol BS8 1TQ, UK

**Keywords:** evolution, behavioural ecology, neuroethology, Lepidoptera, larvae

## Abstract

To evolve, and remain adaptive, collective behaviours must have a positive impact on overall individual fitness. However, these adaptive benefits may not be immediately apparent owing to an array of interactions with other ecological traits, which can depend on a lineage's evolutionary past and the mechanisms controlling group behaviour. A coherent understanding of how these behaviours evolve, are exhibited, and are coordinated across individuals, therefore requires an integrative approach spanning traditional disciplines in behavioural biology. Here, we argue that lepidopteran larvae are well placed to serve as study systems for investigating the integrative biology of collective behaviour. Lepidopteran larvae display a striking diversity in social behaviour, which illustrates critical interactions between ecological, morphological and behavioural traits. While previous, often classic, work has provided an understanding of how and why collective behaviours evolve in Lepidoptera, much less is known about the developmental and mechanistic basis of these traits. Recent advances in the quantification of behaviour, and the availability of genomic resources and manipulative tools, allied with the exploitation of the behavioural diversity of tractable lepidopteran clades, will change this. In doing so, we will be able to address previously intractable questions that can reveal the interplay between levels of biological variation.

This article is part of a discussion meeting issue ‘Collective behaviour through time’.

## 1. Introduction

The ability to live and thrive as part of a group has evolved in many organisms. Decades of research have explored the trade-offs that must be balanced for grouping to be beneficial, the traits that facilitate or constrain the evolution of this behaviour, and the ways in which group cohesion is maintained [1]. The field of collective behaviour is testament to the success of Tinbergen's [2] delineation of behavioural science with clear progress made in understanding subjects outlined in Tinbergen's approaches [2], such as the evolutionary history and adaptive value of such behaviours, as well as the developmentary processes, neural mechanisms and behavioural ‘rules’ that shape group coordination [1]. Yet, despite the flourishing of both evolutionary and reductionist approaches to collective behaviour, these fields remain largely distinct. However, the integration of different methods of understanding phenotypic variation raises new and informative questions about evolutionary processes, such as: what ecological and evolutionary factors determine variation in the mechanistic basis of behaviour? Can the nature of a proximate basis of behavioural variation alter the outcome of selection? Also, does behaviour play a leading role in diversification and adaptation? If a study system can be sufficiently understood within each of Tinbergen's four approaches, the subsequent integration of these fields could help further our understanding of the behaviour overall. To integrate multiple approaches to investigating behavioural traits, some study systems must display a combination of evolutionary diversity, experimental tractability and growing sets of functional tools [3]. Here, we argue that larval Lepidoptera, a long-standing system for studying the evolution of collective behaviour, present one such opportunity.

‘Sociality’ between caterpillars can be difficult to determine, as putative ‘aggregations’—collections of individuals in the same space [4]—may be passive and simply the result of sparsely distributed host plants [5,6]. Collective behaviour instead reflects instances where larvae live and/or feed as a gregarious [7] group, acting in a coordinated fashion to achieve the same goal [8], often including social behaviours such as communication or cooperation between group members [4]. Additionally, levels of larval social complexity differ significantly between species, from patch-restricted systems that involve individuals forming a shelter over the feeding substrate on which they remain throughout larval development, to nomadic foraging larvae which move as a coordinated group between feeding patches, and central-place foragers which build long-term shelters from which they forage [9,10]. In addition to grouping, Lepidoptera display a range of collective behaviours, including trail following [10,11] and synchronized defensive actions [7], which require inter-individual coordination. Allied to these behavioural traits are a huge diversity in morphology, size, host plant ecology and ontogenetic variation. These sources of variation can provide the basis for understanding the evolution and adaptive benefits of social behaviour (e.g. [12]).

Here, initially using Tinbergen's delineation of ethological research [2] as a framework, we highlight the work that has been done to explain the evolution, adaptive benefits, developmental variation and mechanisms maintaining larval collective behaviour in Lepidoptera. We subsequently discuss the potential for this work on larval Lepidoptera to be integrated across behavioural disciplines, and propose future research which aims to link variation across biological levels, providing a more complete understanding of collective behaviour and the interactions between mechanistic and adaptive processes.

## 2. Evolution: repeated shifts to collective behaviour, and associated traits

**Figure 1.**
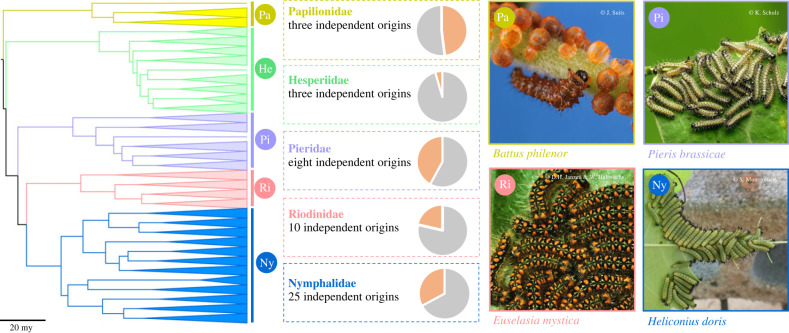
Social behaviour in butterfly (Papilionoidea) larvae: an illustration of the repeated, independent evolution of gregariousness. A genus-level phylogeny of the butterflies from Chazot *et al*. [15], shows only major sub-clades for five families analysed in McLellan *et al*. [12], with Lycaenidae excluded owing to their frequent, derived, larval associations with ant nests. The middle panel illustrates the proportion of gregarious (orange segment of the pie chart) and solitary genera (grey segment), and the number of independent transitions to gregariousness estimated by McLellan *et al*. [12]. To the right, photographs illustrate examples of gregarious larvae from four families; Pa: a first instar *Battus philenor* (Papilionidae) is shown amid a cluster of eggs (image credit: Julia Suits, CC-0), Pi: aggregated *Pieris brassicae* (Pieridae), which uses regurgitation as a behavioural defence [16] (image credit: Katya Schulz CC-2.0), Ri: *Euselasia mystica* (Riodinidae) (image credit: DH Janzen and W Hallwachs, used with permission) and Ny: *Heliconius doris* (Nymphalidae) (image credit: S. Montgomery CC-2.0) both of which exhibit trail following behaviour.

**Figure 2.**
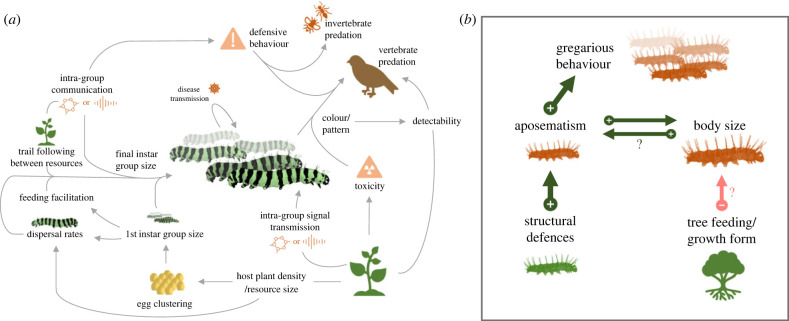
Collective behaviours in eco-evolutionary contexts: (*a*) complexity of proposed interactions between larval behaviour and ecological traits which may shape when/why collective behaviours evolve in Lepidoptera, and mechanisms of intra- and interspecific communication. (*b*) Established evolutionary links between traits demonstrate the restricted extent of our understanding of ecological interactions surrounding group living. The figure illustrates the results of a phylogenetic pathway analyses attempting to reconstruct causative transitions between multiple traits [12] across butterfly larvae. Arrows indicate putatively causative interactions, with positive effects in green and negative effects in red. Question marks indicate results that are sensitive to the dataset used in the analysis. Caterpillar illustrations by Amaia Alcalde.

Although collective behaviour occurs relatively infrequently compared to solitariness, it has evolved repeatedly across Lepidoptera [12–14], and within major clades such as butterflies (e.g. figure 1). This suggests that specific ecological conditions are required to overcome costs associated with grouping, including increased competition for resources [17,18]. While traits such as warning coloration, toxicity and body size have been linked to the evolution of collective behaviour in lepidopteran larvae [12–14,19] (figure 2), the interactions between these traits and other ecological variables are unresolved. This leaves important, unanswered questions about the conditions in which this behaviour arises. For example, by reconstructing the evolutionary history and timing of transitions to group living, we can determine how larval behaviour depends on parental host plant use, e.g. [18], how traits such as warning signals bias the evolution of collective behaviour, or vice versa, and determine how factors such as the spatial distribution, size, foliage and defences of the host plants influence larval behaviour. These questions relate to the dual role of larvae in plant–animal and predator–prey interactions, and the evolutionary impact of these relationships.

Trophic interactions between larvae and their host plants are likely to be an important constraint on the evolution of collective behaviour (figure 2). For example, egg clustering is a strong predictor of larval aggregation [5,18,20], and can be explained by reduced requirements for females to search for patchily distributed host plants [5,6], as well as micro-climactic effects such as reduced risk of egg desiccation [20]. Egg clustering is tightly associated with larval aggregation [21], with all known grouping species resulting from this type of laying pattern [20]. It is, therefore, likely that the selection pressures favouring egg clustering provide the initial opportunity for collective behaviours to evolve [18,20,22]. In addition, while a parental switch to oviposition on sparsely distributed hosts can lead to aggregation [5,6], foliage density, size and the general structure of the plant could support or hinder larval grouping by determining levels of competition and dispersal, and the landscape of predation. Indeed, in some clades, the growth form of the host plant is linked to associated traits such as aposematism [23,24], and collective behaviour may be less likely to evolve in tree-feeding species where potential egg laying areas are high [12].

However, not all larvae which hatch from clustered eggs are gregarious [20], suggesting additional, independent selection pressures must shape larval behaviour and communication mechanisms. In part, these selection pressures relate to anti-predator defences. Most notably, larval grouping and aposematism (the coupling of a warning signal with a chemical defence) frequently co-occur [14], although the evolutionary order in which these two traits are typically acquired has been debated [14,25,26]. By extension, a transition to feeding on a toxic host, and thus acquiring a toxic defence, could be an important step towards aggregated larvae [6]. This causal relationship remains understudied, but in most cases, toxicity is probably acquired before a warning signal and/or gregariousness [13,14,26,27], and recent evidence provides strong support for the evolution of a warning signal evolving prior to, and potentially driving, the subsequent evolution of collective behaviour [12,19], which in turn amplifies the warning signal [28–30] (figure 2). Larval body size may also influence the evolution of collective behaviour, again by amplifying warning signals [12,17], or by aiding group survival through faster satiation of predators [31]. However, competition for resources between grouped individuals may impose a trade-off between body size and group size, as females are limited in the resources that they can allocate to each egg to ensure larger larvae [17]. A recent comparative study across butterflies found no evidence supporting a direct evolutionary interaction between final instar size and grouping, instead suggesting that body size probably increases before the evolution of collective behaviour, mediated by the acquisition of a warning signal as crypsis becomes less effective with increased size [12].

The results of comparative analyses have, therefore, identified repeated phylogenetic origins of larval collective behaviour, as well as other traits with which it co-evolves, revealing potential causative ecological interactions. They also reveal how the wider ecological context, and evolutionary history, of a species will determine the likelihood and expression of collective behaviours. However, less is known about the potential influence of larval host plant use on the evolution of collective behaviour. Given that the spatial distribution, foliage density and chemical defences of hosts might affect the benefits of aggregating, this deserves considerably more attention.

## 3. Adaptation: fitness benefits of anti-predator defences in social caterpillars

Directly studying the fitness effects of alternative behavioural strategies can provide a fuller understanding of the conditions in which a behavioural trait evolves, and test adaptive predictions from comparative studies of a trait's evolutionary history. By aggregating, larvae can gain many advantages, such as enhancing individual foraging success [32–34], faster development [33,35,36], improved thermoregulation [4] and facilitated nest construction [4,37]. However, one of the most well-established advantages of aggregation is an enhanced protection against predators [7,11,18]. Grouping can reduce the chance of a given individual being singled out by a predator, an effect which is enhanced when coupled with the added deterrence of aposematism [11,31]. Warning coloration is common across species that exhibit collective behaviour [11,17], but often only appears in later instars, with crypsis being the dominant strategy during early instars [38–41]. Colour pattern may also improve the conspicuity and/or memorability of the signal [42], yet relatively few studies have tested for pattern-based differences in warning signal enhancement [42] and fewer still have looked for this effect in aggregated prey [43].

However, not all gregarious species have a warning signal, and these case studies provide further insight into how groups balance the increased detectability costs that come with grouping. First, in some contexts, predators may learn that aggregation itself can be a signal for unprofitability [30,44]. Second, defensive behaviours such as thrashing, regurgitation of noxious fluids and head flicking are also effective predator and parasitoid deterrents [7]. Indeed, the protective effect of such behaviours may be restricted to group settings [45,46], raising the prospect that the evolution of collective behaviours could favour the evolution of secondary social defence behaviours. Regardless, aggregated larvae are able to individually perform costly defensive behaviours less frequently, while benefitting from the protection of their group [16,47,48]. Third, one basic anti-predator benefit of group living is the protection offered by the dilution effect [49,50], where individuals have a lower probability of being singled out by a predator. Protection via dilution is also enhanced when prey are defended, as experienced predators often avoid the group [12,14,30,31]. In addition to dilution, threatened prey can decrease the relative likelihood of being attacked by positioning themselves close to the centre of the group [51], which Hamilton described as the ‘selfish herd’ effect [52]. For example, in tent caterpillar (*Malacosoma disstria*) aggregations, those nearer the centre of the group are better protected from attack from spiders and hemipterans [53], possibly because these predators attack along a lateral plane and encounter peripheral individuals first. However, peripheral larvae are also at greater risk of attack from parasitoid wasps [53,54], which do not necessarily approach laterally. Finally, even in the absence of top-down biotic factors, plant–herbivore interactions may favour grouping, if collectively overcoming leaf defences permits access to greater resources and faster development times [18,32,33].

Quantification of relative fitness benefits of grouping in different contexts again reveals the impact of ecological interactions (figure 2), with the degree and type of predation impacting what traits affect the fitness consequences of gregariousness. This is true for morphological traits such as patterning, defensive behaviours, individual movements and positioning within groups, all of which may depend on the behavioural strategies of dominant predators. However, it is currently unclear whether some defensive traits, such as aposematic colour patterns, structural defences and defensive behaviours, provide additional benefits when in a group setting compared to when solitary. As such, the extent to which group living alters the selection regimes acting on other traits is underexplored. While our focus here has been on anti-predator traits, we also note effects of group living may extend to physiology and immunity.

## 4. Development: stage-dependent selection and behavioural switches

Selection pressures often change for organisms as they develop, such that a strategy which is beneficial early in life can become disadvantageous in later stages. Many lepidopteran larvae exhibit a clear behavioural switch during development, transitioning away from collective behaviours in later instars [55–57]. This raises two central questions, why do some species retain their collective behaviour up to pupation while others transition away from it? Also, why is collective behaviour so much more common in earlier instars?

First, egg clustering may again play a role in the initial aggregation of larvae [5,18,20]. Passively aggregated larvae may then disperse at different stages, or not at all. Many non-dispersing species are reported to live in highly social, cohesive communities, often within silk nests [58–61]. In these contexts, there is limited benefit to individuals in dispersing, especially when later instars still benefit from feeding facilitation (e.g. [58]), are under threat from parasitoids [45,62] or when risky search expeditions for new host plants are performed as a group (e.g. [60]).

Second, outside these highly specialized cases where larvae share communal nests and foraging trails, young larvae may also benefit from transient advantages to the group. Young larvae may struggle to overcome leaf defences, and rely on feeding facilitation from conspecifics [18,33,34,36,63] (figure 2). For example, older *Uresiphita reversalis* larvae can better overcome plant defences, making it easier for younger larvae to feed [34], and the collective attack of a leaf can be enough to overcome its defences [36,63]. Group feeding can result in faster development for young larvae [33,35,36], allowing them to escape the vulnerable early instars more quickly [45,64,65]. Conversely, older, and necessarily larger, larvae are more likely to overcome plant defences on their own [33,34], can better defend themselves against invertebrate enemies [56,64], and experience increased competition between conspecifics [18,56,57,66]. Dispersal might also be a response to the high detection costs of unprotected larvae as they grow larger and become more active [23,67]. If this is the case, variation in species’ ecologies should explain differences in ontogenetic changes in behaviour. For example, two allopatric subspecies of *Byasa alcinous* larvae exhibit host plant-mediated variation in behavioural phenotype [32]; one feeds on a well-defended host plant and forms large groups, whereas the other's host has comparatively softer leaves and it forms smaller groups or is occasionally solitary [32]. This variation may also be environmentally determined, consistent with evidence that some larvae can plastically vary their group size, instar number, instar length and overall development time in response to a number of abiotic, biotic and physiological factors [48,59,68–71].

These data suggest two non-mutually exclusive modes of altering collective behaviour, motivation to disperse from a feeding patch and group attraction. In some species, early instar aggregation may be primarily explained by low dispersal, but in others, particularly in trail following species, group attraction clearly plays a role. These two factors could, in theory, interact to alter group size, as dispersing individuals could form splinter groups when attraction remains high. Regardless, while late instar gregariousness is probably a derived behaviour [12,13,19], less is known about the evolution of ontogenetic behavioural switches in collective behaviour.

Currently, very little work has been dedicated to identifying specific developmental mechanisms associated with the ontogenetic shift from aggregation to solitariness. Recently, the first steps towards identifying genes controlling developmental switches were taken using *Drepana arcuata*, which transitions away from gregariousness in the third instar [72]. Using a time series of gene expression profiles, a set of genes with differential expression between solitary and grouped instars was identified. Although this gene set probably includes stage-dependent changes unrelated to the behaviour, subsequent functional analysis of one candidate gene, the octopamine receptor gene (*DaOAR*), suggests that expression levels of this protein alter larval gregariousness [73]. *DaOAR* is linked to changes in group behaviour in other insects [74,75], suggesting convergent mechanisms underpinning developmental transitions in collective behaviours. To our knowledge, this represents the first step in uncovering the genetics behind larval collective behaviour in Lepidoptera, providing a foundation for future comparative and mechanistic analyses.

## 5. Mechanism: multiple routes to group coordination

Regardless of how collective behaviour evolves, truly social species need ways of maintaining group cohesion, particularly as increasing plant consumption necessitates wider foraging movements. Communication between group members is often a vital component of successful group living. Much of the research on communication between larval Lepidoptera has focused on the tent caterpillars in the *Malacosoma* genus [67,76], which live together in highly social nests and communicate the locations of food patches to group members using pheromone-laden silk trails [9]. Much less is known about the communication methods of other species exhibiting patch-restricted social structures [9]. Nevertheless, communication via pheromone-laden silk trails appears to be a common mechanism in species with varying levels of sociality [9,67,77]. In most cases, only the pheromone, not the silk, elicits the following behaviour [76,78]. Larvae may also discern various properties of this chemical signal, such as its age, strength, whether it was laid by a conspecific and whether the trail-maker successfully located a feeding patch [78–80]. Trail pheromones may also be conserved between close relatives, facilitating mixed-species groups [81]. Some nomadic foraging species primarily use tactile cues when trail following, but often also lay down pheromones to aid disconnected members to re-join the procession [82]. Interestingly, however, some trail following species, such as *Mechanitis menapis*, do not appear to produce any trail pheromones, and instead use silk to facilitate patch-restricted grouping [83]. This suggests that at least some species which form groups with lower social complexity than in nomadic forager and central-place systems may be less reliant on communication through pheromones.

A second mode of communication commonly used by larval Lepidoptera is vibrational signalling, which can convey a range of information including during territorial disputes [84–86], communication with associated ants and social interactions with conspecifics [85–87]. However, evidence for vibrational communication between conspecifics of group-living larval Lepidoptera is limited [85,88]. One of the only recorded examples of this behaviour involves *D. arcuata*, which form small, patch-restricted groups during their early instars [85]. *Drepana arcuata* do not appear to attend to chemical cues left by conspecifics, and primarily rely on vibrations to recruit conspecifics to their nest [85].

There is even less evidence for the use of auditory signalling between caterpillar conspecifics. While the larvae of some species produce airborne sounds in response to perceived predation threats [87,89,90], in most cases these signals are directed at the predator [87]. However, in response to predator threat stimuli, *Hylesia nigricans* larvae produce ultrasonic emissions that are outside the hearing range of their predators, and which elicit anti-predator behaviour in group members [88]. This suggests auditory communication may be used by at least some social species.

Given the restricted number of case studies available, it is difficult to draw general conclusions about the mechanisms by which independently derived collective behaviours are maintained and coordinated across Lepidoptera. In addition, very little is known about how ancestral sensory ecologies and environmental contents impact the efficiency of different communication mechanisms. For example, the costs of active communication are poorly explored, while the distribution of vibrational communication could be limited by the transmission properties of host plants [91], and chemical cue efficiency can be impacted by wider environmental conditions [92]. This area, therefore, provides excellent opportunities for novel research seeking to explore these effects.

## 6. Future directions: towards an integrative understanding of collective behaviour

To date, the extensive variation in larval forms and behaviours across the Lepidoptera have been used to test and develop evolutionary hypotheses about the costs and benefits of alternative behavioural strategies, and their dependency on other traits, although much less work exists on the developmental and mechanistic basis of variation in larval behaviour, Lepidoptera are well suited for these kinds of studies, and for studies which integrate ‘ultimate’ and ‘proximate’ mechanisms to bridge traditional boundaries of behavioural biology. First, the well characterized phylogenetic relationships across Lepidoptera [15,93], coupled with the repeated, independent origin of collective behaviours [12–14,19] (figure 1) provide a powerful basis for comparative analyses at multiple biological levels, from the molecular to the behavioural [94]. Second, allied to a history of interest in phylogenetic studies, Lepidoptera already have a wealth of genomic resources available [95,96], which, together with the development of tools to manipulate candidate genes (e.g. [97]), provide a basis to study the genetic and developmental variation in behavioural traits. Of particular interest will be closely related species with divergent behavioural traits, where the confounding effects of phylogenetic and ecological distance may have less impact. Finally, we note that developments in behavioural tracking of unmarked individuals [98] will make manipulating and quantifying group behaviours more tractable. With this developing experimental and comparative toolkit, we suggest larval Lepidoptera can be used to explore the integrative biology of collective behaviour. Below, building on our discussion above, we highlight examples of questions that can emerge by taking this approach.

### (a) How do host plant ecologies impact the evolution of larval behaviour?

Properties of larval host plants probably shape both the evolutionary trajectories of larval behaviour and the mechanisms that underpin this variation. Comparisons of host plant traits between larvae of closely related species which differ in grouping behaviour could provide insights into both questions. This will reveal whether host plant spatial distributions, size or toxicity are important in promoting collective behaviour; while comparisons of the physical properties of leaf/stem material, coupled with behavioural assays such as for vibrational communication, may reveal whether host plants determine the communication landscape for maintaining group cohesion.

### (b) How does variation in selection regimes across larval stages shape the evolution of developmental switches in behaviour?

Accumulating data on ontogenetic shifts in gregariousness across species would facilitate comparative analyses to determine whether or not these shifts were present ancestrally, whether they reflect an intermediate state to consistent larval gregariousness, and how related traits such as coloration and size covary across developmental and evolutionary time. Phylogenetically independent case studies of species with ontogenetic behavioural shifts can be used to test whether these developmental effects share a common molecular basis, or if developmental processes are reshaped through independent mechanisms.

### (c) Do changes in responsiveness to conspecific cues occur at the sensory periphery or in downstream circuits?

Characterizing the mechanisms used by gregarious species to coordinate their movement will also facilitate assays of how related solitary species respond to these cues. Coupled with comparative genomics, or transcriptomics, it may be possible to identify molecular, and subsequently neural, pathways which diverge between species, and begin to understand the relative frequencies at which either changes in sensory attention to conspecific cues, or changes in the way these cues are processed in the central brain, determine group coordination.

### (d) Do similar mechanisms underpin evolutionarily convergent shifts in collective behaviour, and is this determined by similarities in ecology?

While some collective behaviours may be outwardly very similar, whether or not they are coordinated by similar mechanisms remains unclear. This is important because this variation may shape the efficiency or limitations of these behaviours in a natural context, and could provide insights into the relative roles of different ecological contexts, evolutionary histories and sensory ecologies in determining the outcome of selection for increased collective behaviours.

### (e) To what extent are behavioural shifts constrained by both intrinsic and extrinsic processes?

Ultimately, understanding both the evolutionary selection pressures that shape behavioural evolution, and the intrinsic mechanisms that produce variation, permits more robust assessments of the interplay between these biological levels, and the hypothesis that phenotypic evolution is unconstrained by proximate mechanisms (the ‘phenotypic gambit’ [39]). While not possible at large phylogenetic scales, selecting case studies that deviate from general patterns using comparative analyses may provide insights into whether these exceptions evolve owing to peculiar ecological contexts or mechanistic constraints.

While these questions reflect major challenges, we have the foundations to work towards answering them. We stress that identifying tractable variation and establishing a clear understanding of a behavioural trait and its ecological relevance, including distinguishing active grouping (either behaviourally or evolutionarily) from passive aggregation, remains a necessary *a priori* to understanding its proximate basis. With improvements in the power of phylogenetic analyses, further development of methods that permit the accurate quantification and/or manipulation of behavioural traits, and the ability to associate these traits with genetic variation, we argue that larval Lepidoptera are well placed to contribute to the emergence of new, integrative case studies in the evolution of collective behaviour.

## Data Availability

This article has no additional data.
